# 1493. Targets for Primary Prevention in Newly Diagnosed People Living with HIV: Applying *Life's Simple 7* to Assess Cardiovascular Health Needs in ART-Naive People Living with HIV in Tanzania

**DOI:** 10.1093/ofid/ofad500.1328

**Published:** 2023-11-27

**Authors:** Safah Khan, Gloria J Manyangu, Robert N Peck

**Affiliations:** Weill Cornell Medicine-Qatar, Maryland; Bugando Medical Centre, Mwanza, Tanzania, Mwanza, Mwanza, Tanzania; Weill Cornell Medical College, New York City, New York, USA, New York, New York

## Abstract

**Background:**

People living with HIV (PLWH) are at elevated cardiovascular disease risk by virtue of their infection, even before initiation of antiretroviral therapy (ART). There is a need to characterize cardiovascular health (CVH) during the early infection phase to appropriately direct primary prevention efforts. Therefore, we applied American Heart Association's *Life's Simple 7 (LS7)* scale to compare CVH between ART-Naive PLWH and HIV-uninfected adults in Tanzania.

**Methods:**

A cross-sectional analysis was conducted on a cohort of ART-Naive PLWH and HIV-uninfected adults recruited from HIV clinics between June 2016 and August 2019 in Mwanza, Tanzania (HIV&HTN cohort). We applied modified *Life's Simple 7 (LS7)* definitions (**Table 1**), categorizing each metric as ideal (2 points), intermediate (1 point) and poor (0 point). We compared distribution of these categories for each *LS7* metric between study groups. Ordinal regressions were employed to investigate associations between HIV status and each individual *LS7* metric adjusting for age, sex, education, income.

**Figure:**
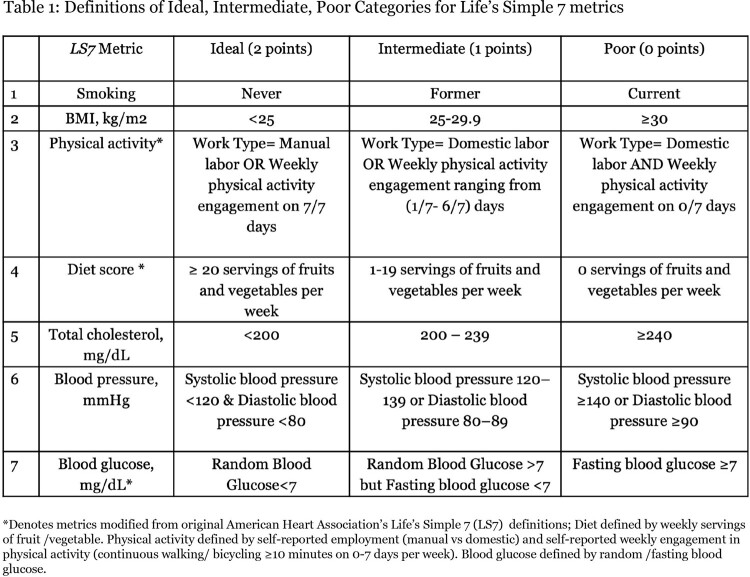

**Results:**

Our study included 997 participants (493 PLWH, 504 controls). The distribution of *LS7* metrics differed between PLWH and HIV-uninfected controls, as displayed in **figures 1a** and **1b**. PLWH had higher prevalence of ideal BMI (75.6% vs 65.4%) and ideal blood pressure (56.1% vs 35.1%) than HIV-uninfected counterparts. By contrast, PLWH had lower prevalence of ideal smoking (83.5% vs 89.6%), physical activity (38.9% vs 45.0%), blood glucose (93.9% vs 97.8%) compared with controls. These associations were validated by multivariate ordinal regression wherein PLWH were found to be more likely to have ideal BMI (aOR= 1.59 [95%CI= 1.20-2.12], p=0.001), ideal blood pressure (aOR =2.50 [1.95-2.31], p< 0.001) but less likely to have ideal smoking (aOR =0.63 [0.49-0.97], p=0.04), ideal physical activity (aOR= 0.63 [0.49-0.81], p< 0.001), ideal blood glucose (aOR=0.34 [0.16-0.68], p=0.003).

**Figure:**
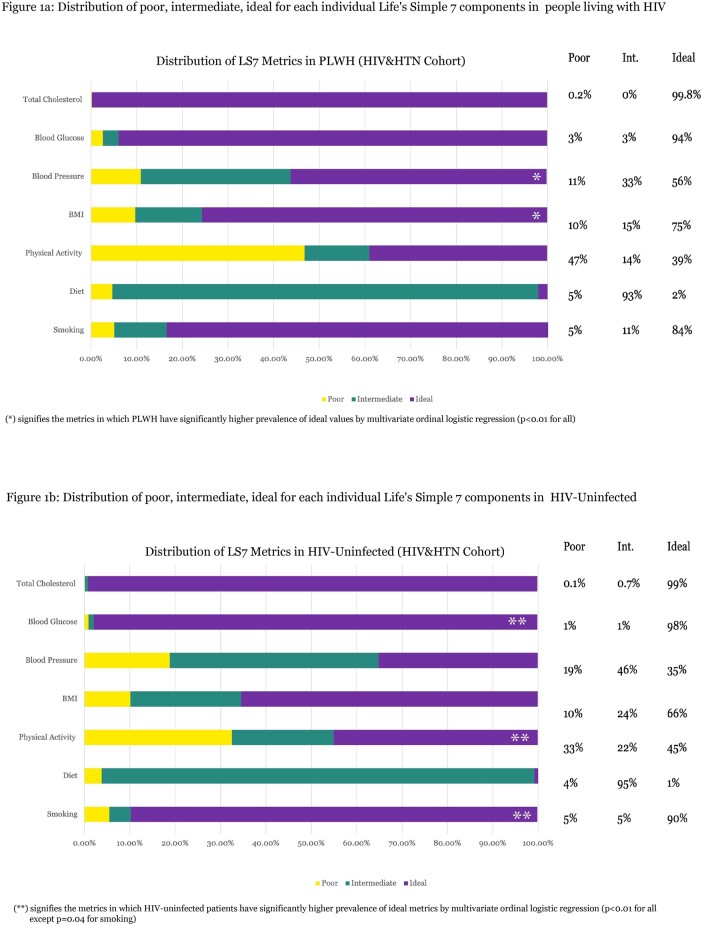

**Conclusion:**

Cardiovascular health profiles differ between PLWH and uninfected counterparts, even in the early pre-ART phase. Although PLWH had lower rates of traditional risk factors, there is room for improvement with regards to smoking cessation, improving physical activity and diabetes screening in newly diagnosed PLWH.

**Disclosures:**

**All Authors**: No reported disclosures

